# Supplementary figures and images for: The FUR-like regulators PerRA and PerRB integrate a complex regulatory network that promotes mammalian host-adaptation and virulence of Leptospira interrogans
Source: PLoS Pathog. 2021 Dec 2;17(12):e1009078. doi: 10.1371/journal.ppat.1009078 (PMC8638967; doi:10.1371/journal.ppat.1009078)

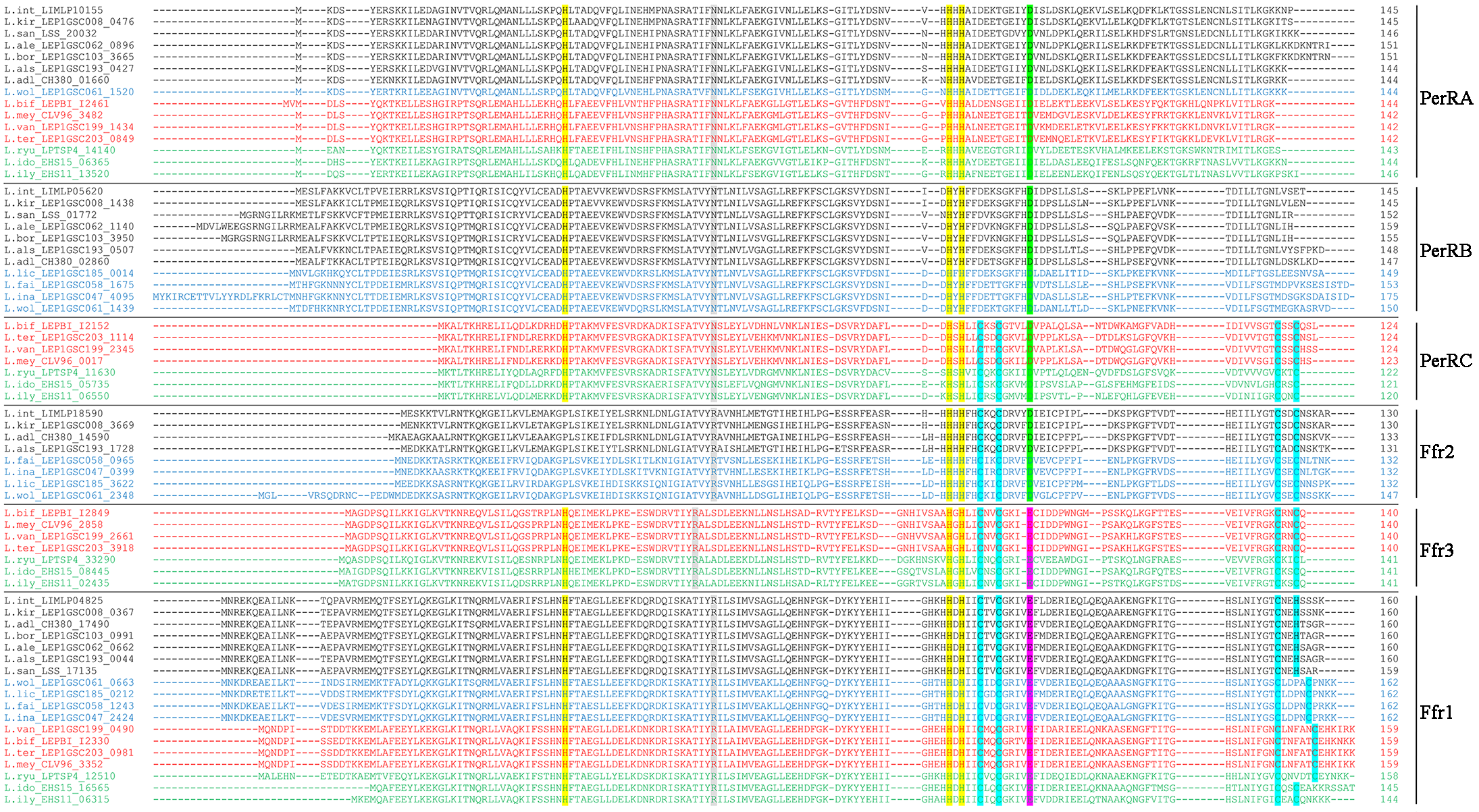

Supplement: S1 Fig — Species from pathogenic subclades P1 and P2 are colored in black and blue, respectively, while saprophytic species from subclades S1 and S2 are in red and green, respectively. Genomic locus tags for each FUR family proteins in Leptospira spp. are indicated. Highly conserved residues predicted as regulatory metal binding sites are highlighted in yellow, green and magenta. Putative structural metal binding sites are highlighted in cyan. Leptospira species are abbreviated as follow: L. int, L. interrogans; L. kir, L. kirschneri; L. adl, L. adleri; L. als, L. alstonii; L. san, L. santarosai; L. bor, L. borgpetersoni; L. ale, L. alexanderi; L. wol, L. wolfii; L. lis, L. liscerasiae; L. ina, L. inadai; L. fai, L. fainei; L. bif, L. biflexa; L. mey, L. meyeri; L. ter, L. terpstrae; L. van, L. vanthielii; L. ryu, L. ryugenii; L. ily, L. ilyithenensis; L. ido, L. idonii. (TIF) [file ppat.1009078.s001.tif]

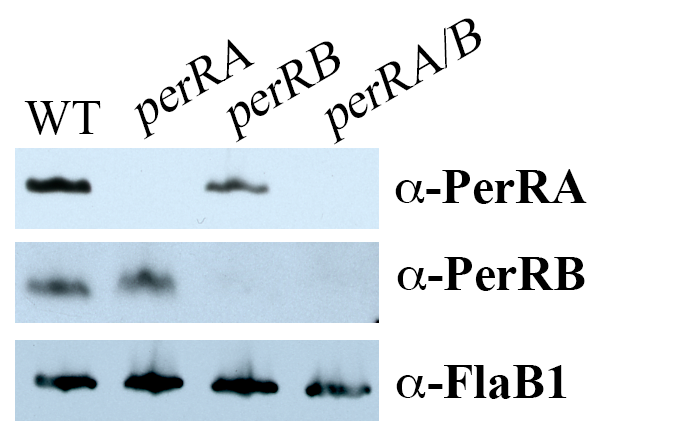

Supplement: S2 Fig — Whole cell lysates of L. interrogans sv. Manilae strain L495 wild-type (WT), perRA, perRB and perRA/B strains cultivated in vitro. Lysates were separated by SDS-PAGE, transferred to nitrocellulose, and probed with rat polyclonal PerRA- or PerRB-specific antiserum. Membranes were stripped and re-probed using rat polyclonal antiserum against recombinant FlaB1 as a loading control. (TIF) [file ppat.1009078.s002.tif]

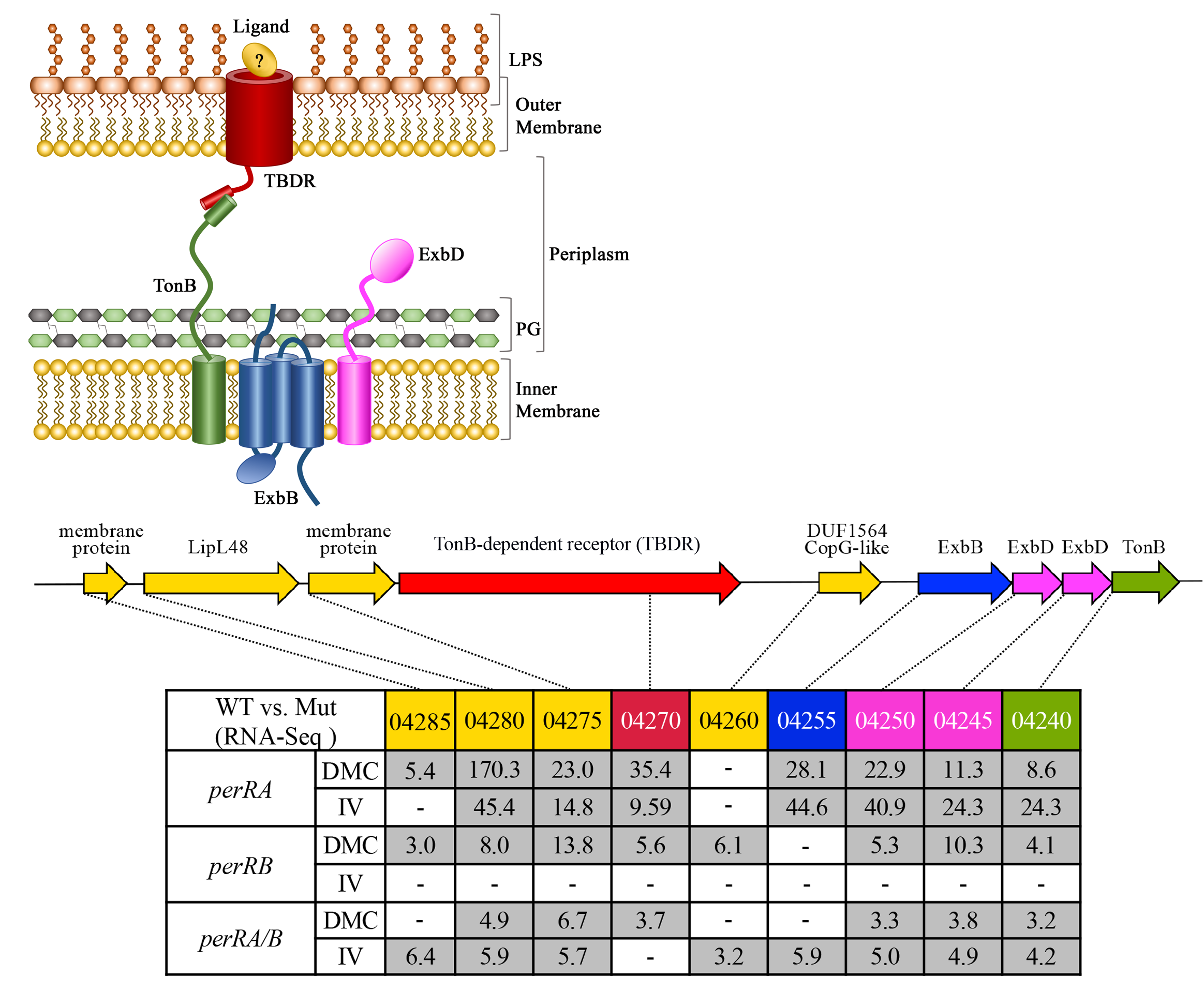

Supplement: S3 Fig — Data from comparative RNA-Seq analysis of wild-type (WT) vs. perRA, perRB and perRA/B strains identified a nine gene chromosomal locus that includes lipL48 and genes encoding a TonB-dependent receptor and ExbB/ExbD/TonB transporter. Fold-of-regulation for each gene are based on RNA-Seq data from wild-type and mutant leptospires grown in DMCs, presented in S2–S4 Tables, and in vitro in EMJH at 30°C (IV), presented in Zavala-Alvarado et al. [93, 94] (TIF) [file ppat.1009078.s003.tif]

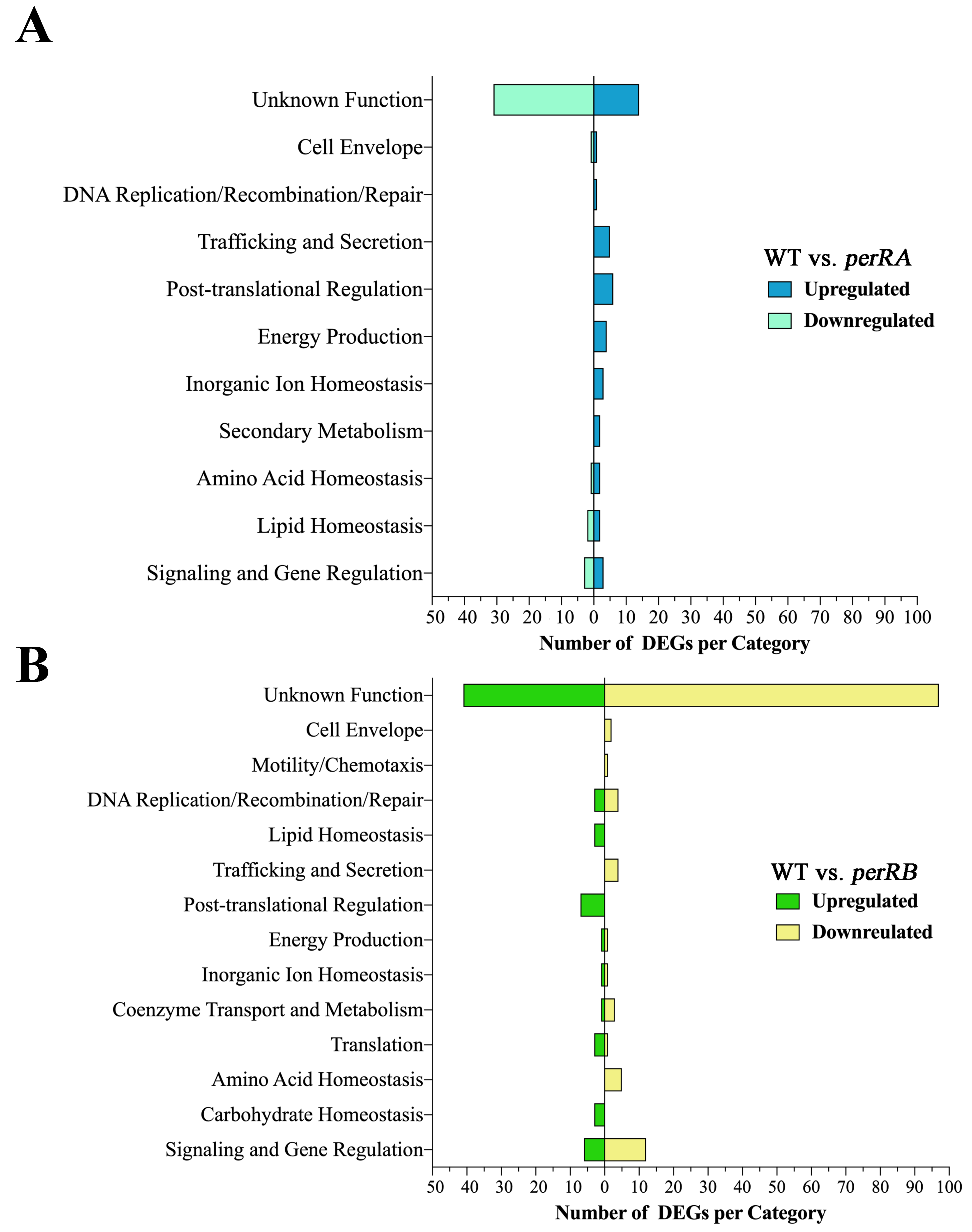

Supplement: S4 Fig — Cluster of Orthologous Genes (COG) categorization of differentially expressed genes (DEGs) in the wild-type (WT) vs. perRA (A) and perRB (B) RNA-Seq comparisons. COG predictions for individual genes are presented in S2 and S3 Tables. Number of DEGs in each COG are indicated on the x-axis. (TIF) [file ppat.1009078.s004.tif]

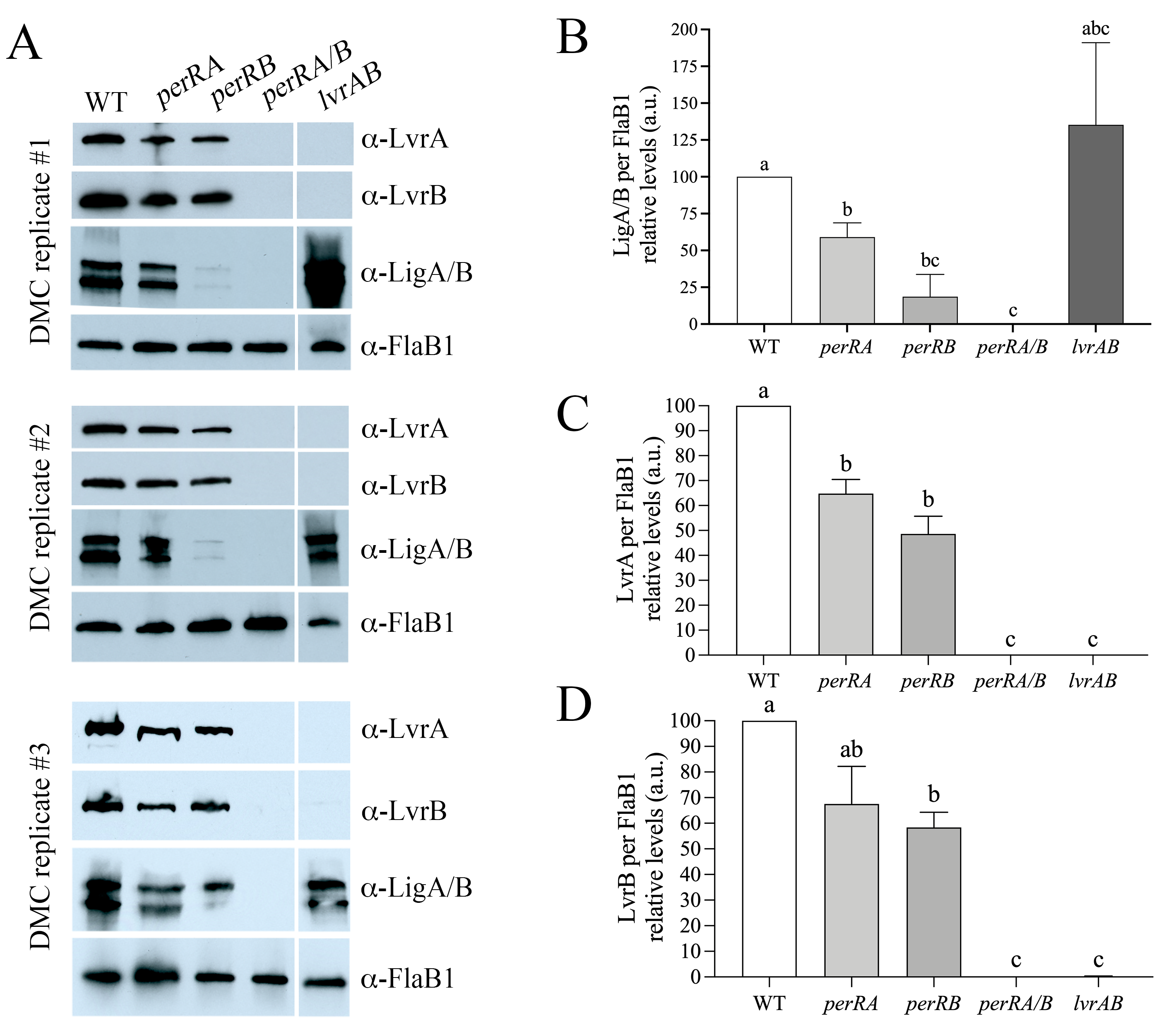

Supplement: S5 Fig — A. Whole cell lysates of L. interrogans sv. Manilae strain L495 isogenic wildtype (WT), perRA, perRB, perRA/B and lvrAB strains were generated from leptospires cultivated within DMCs, separated by SDS-PAGE, probed with polyclonal antiserum against LvrA, LvrB, or N-terminal conserved repeat region for LigA/LigB. Panels represent independent biological replicates and detected by chemiluminescence imaging as described in Methods. After detection, membranes were stripped and re-probed using polyclonal antiserum against recombinant FlaB1 as a loading control. Intensity values for LigA/B (combined), LvrA, and LvrB in each replicate were quantified using ImageJ and the normalized based on values for FlaB1 in the same lysate. Normalized values for mutant strains were compared to those from the WT, which was set to 100. Bars represent the standard error of the mean from three biological replicates. Significant was determined in Prism (GraphPad) using a two-tailed t-test. Different letters indicate a significant difference (p ≤ 0.05) in pairwise comparisons. (TIF) [file ppat.1009078.s005.tif]

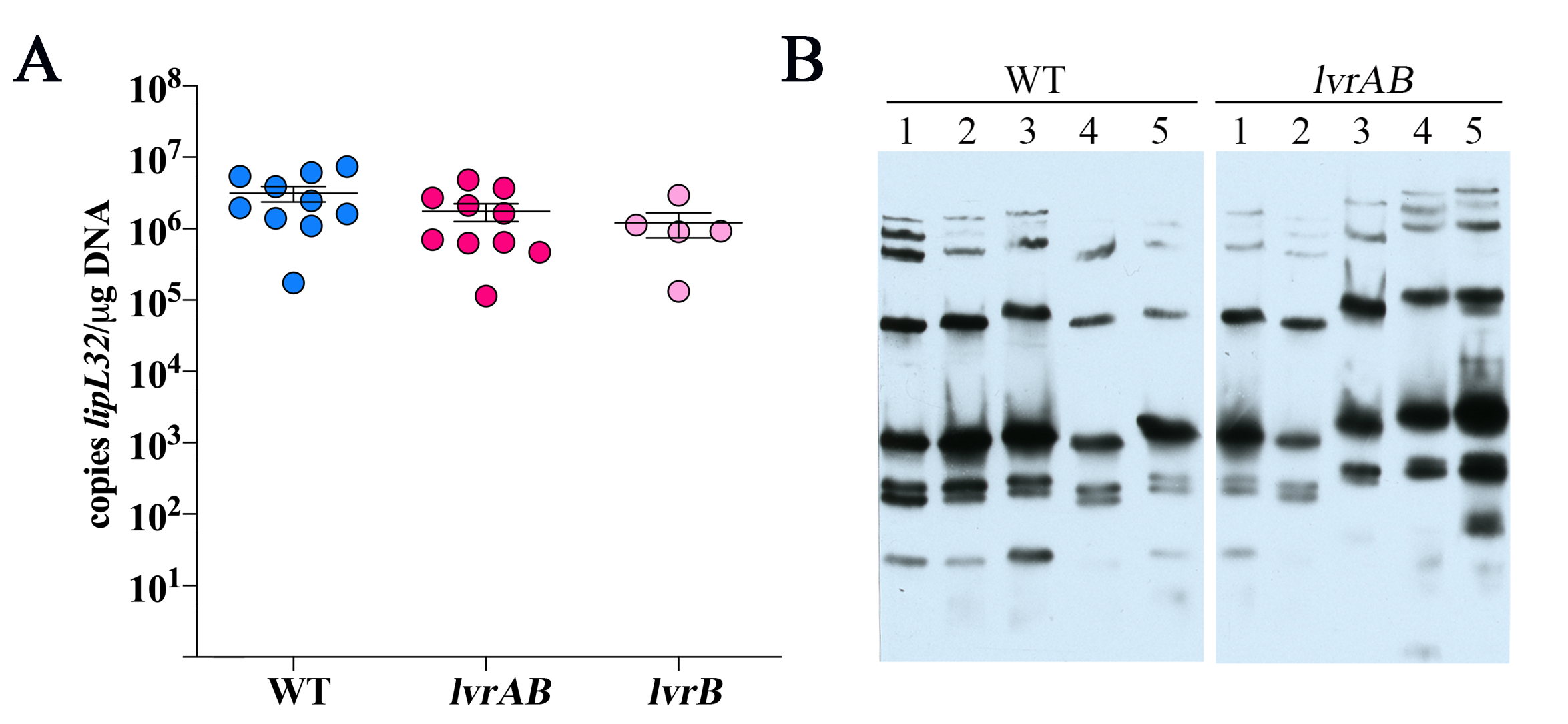

Supplement: S6 Fig — A. Burdens of leptospires in kidneys harvested from mice in Fig 8B. DNA samples from kidneys harvested 28 days post-inoculation were assessed (in quadruplicate) by qPCR using a Taqman-based assay for lipL32. Bars represent the average and standard error of the mean. p-values were determined by comparing burdens in mice infected with wild-type (WT) and mutant strains at the same timepoint using a two-tailed t-test; we saw no significant difference (p>0.05) between burdens between the WT, lvrAB and lvrB strains. B. Immunoblot analysis of sera collected from C3H/HeJ mice 28-days following intraperitoneal inoculation with 105 wild-type or lvrAB mutant strains and then used to probe whole cell lysates of L. interrogans sv. Manilae strain L495 grown in EMJH at 30°C. (TIF) [file ppat.1009078.s006.tif]
