# Supplementary material for: The FUR-like regulators PerRA and PerRB integrate a complex regulatory network that promotes mammalian host-adaptation and virulence of Leptospira interrogans
Source: PLoS Pathog. 2021 Dec 2;17(12):e1009078. doi: 10.1371/journal.ppat.1009078 (PMC8638967; doi:10.1371/journal.ppat.1009078)
Supplement: S5 Table — (DOCX) [file ppat.1009078.s011.docx]

**S5_Table. Strain used in these studies.**

| Strain Name | Description | Antibiotics Resistance | Reference |
| --- | --- | --- | --- |
| WT | *L. interrogans* sv. Manilae st. L495, wild-type parent | - | [1] |
| *perRA* | *perRA*::Km^R^, *Himar1* Tn insertion at position 62 bp in *LIMLP10155* | Kanamycin | [1] |
| *perRB* | *perRB*::Km^R^, *Himar1* Tn insertion at position 287bp in *LIMLP05620* | Kanamycin | [1] |
| *perRA/B* | *perRB*::Km^R^ Tn mutant containing an insertion within *perRA*, generated by allelic replacement | Kanamycin, Spectinomycin | [2] |
| *perRA/B+perRB* (*comp*) | *perRA/B* double mutant complemented in *trans* with a wild-type copy of *perRB* (LIMLP05620) expressed under its native promoter on pMaORI-GentR | Kanamycin, Spectinomycin and Gentamycin | [2] |
| *lvrB* | *lvrB*::Km^R^, *Himar1* Tn insertion in *lvrB*/LIMLP08485 | Kanamycin | [3] |
| *lvrAB* | *lvrAB*::Km^R^, *Himar1* Tn insertion in *lvrA*/LIMLP08490 | Kanamycin | [3] |

**References**

1. Murray GL, Morel V, Cerqueira GM, Croda J, Srikram A, Henry R, et al. Genome-wide transposon mutagenesis in pathogenic *Leptospira* species. Infect Immun. 2009;77(2):810-6. Epub 2008/12/03. doi: 10.1128/IAI.01293-08. PubMed PMID: 19047402; PubMed Central PMCID: PMC2632054.

2. Zavala-Alvarado C, Vincent AT, Sismeiro O, Legendre R, Varet H, Bussotti G, et al. The oxidative stress response and virulence of pathogenic *Leptospira* are controlled by the interplay of two peroxide stress regulators. bioRxiv. 2020:2020.11.06.371039. doi: 10.1101/2020.11.06.371039.

3. Adhikarla H, Wunder EA, Jr., Mechaly AE, Mehta S, Wang Z, Santos L, et al. Lvr, a signaling system that controls global gene gegulation and virulence in pathogenic *Leptospira*. Frontiers in cellular and infection microbiology. 2018;8:45. Epub 2018/03/31. doi: 10.3389/fcimb.2018.00045. PubMed PMID: 29600195; PubMed Central PMCID: PMCPMC5863495.
