# Supplementary material for: The FUR-like regulators PerRA and PerRB integrate a complex regulatory network that promotes mammalian host-adaptation and virulence of Leptospira interrogans
Source: PLoS Pathog. 2021 Dec 2;17(12):e1009078. doi: 10.1371/journal.ppat.1009078 (PMC8638967; doi:10.1371/journal.ppat.1009078)
Supplement: S6 Table — (DOCX) [file ppat.1009078.s012.docx]

**S6_Table. Oligonucleotide primers used in these studies.**

| Primer | Sequence (5’-3’) | Purpose | Reference |
| --- | --- | --- | --- |
| lipL32-F | TTGGATCCGTGTAGAAAGAATGTC | qPCR/qRT-PCR | [1] |
| lipL32-F | TCGTCCAATTTTTGAACTGGTTT | qPCR/qRT-PCR | [1] |
| lipL32 probe | [6FAM] CCAAATCGCCAAAGCTGCGAAAGC[BHQ1] | qPCR/qRT-PCR | [1] |
| PerRA-F | AACGCTTCTCGTGCCACTAT | qRT-PCR | This study |
| PerRA-R | CGCGTGATGATGATGGACTA | qRT-PCR | This study |
| PerRB-F | TTTCCTGTTTGGGAAAATCG | qRT-PCR | This study |
| PerRB-R | CCAGAAATTCAGGTGGGAGT | qRT-PCR | This study |
| LIMLP04825-F | ACAGTCTGCGGAAAAATCGT | qRT-PCR | This study |
| LIMLP04825-R | TGTTCGTTGCAAGTTCCGTA | qRT-PCR | This study |
| LIMLP18590-F | CATCTGATGGAAACGGGAAC | qRT-PCR | This study |
| LIMLP18590-R | CACGGTCGCATTGTTTACAG | qRT-PCR | This study |
| Tn5'out | CCGAAGTTCCTATACTTTCTAGAGAATAGGA | Sequencing | This study |
| Tn3'out | AAGCTTTAACTACAAGCTTTTTAGACATCTAATC | Sequencing | This study |
| PerRAseq-F | ATGAAGGATTCTTACGAAAGAAGC | Sequencing | This study |
| PerRAseq-R | TTATGGATTTTTTTTGCCTTTGAGTGTAATG | Sequencing | This study |
| PerRBseq-F | ATGGAATCGTTATTTGCTAAAAAAGTTTGC | Sequencing | This study |
| PerRBseq-R | TTAAGTTTCTGAAACCAGATTTCCGGTAAG | Sequencing | This study |
| PerRAout-F | TCAAATTCTTTAAAATTAAAAATTTATTTC | Sequencing | This study |
| PerRAout-R | AAAACTTAGGTTTTTCTGTAAAATGATG | Sequencing | This study |
| PerRA/pET28a-F | CGCGCGGCAGCCATATGAAGGATTCTTACGAAAGAAGCAAAA | Cloning | This study |
| PerRA/pET28a-R | GTCATGCTAGCCATATGTTATGGATTTTTTTTGCCTTTGAGT | Cloning | This study |
| PerRB/pET28a-F | CGCGCGGCAGCCATATGGAATCGTTATTTGCTAAAAAAGTTT | Cloning | This study |
| PerRB/pET28a-R | GTCATGCTAGCCATATGTTAAGTTTCTGAAACCAGATTTCCG | Cloning | This study |
| LvrA/pET28a-F | CGCGCGGCAGCCATATGATAAATTCAACCGATTTAAAACACA | Cloning | This study |
| LvrA/pET28a-R | GTCATGCTAGCCATATGTTATATCGTTTCTAAAAATGTCCT | Cloning | This study |
| LvrB/pET28a-F | CGCGCGGCAGCCATATGAATAAATGGAAATTCCTTTTCTTAG | Cloning | This study |
| LvrB/pET28a-R | GTCATGCTAGCCATATGTTATTGAACACTTGGGTCG | Cloning | This study |

Literature Cited

1. da Cunha CEP, Bettin EB, Bakry A, Seixas Neto ACP, Amaral MG, Dellagostin OA. Evaluation of different strategies to promote a protective immune response against leptospirosis using a recombinant LigA and LigB chimera. Vaccine. 2019;37(13):1844-52. doi: 10.1016/j.vaccine.2019.02.010. PubMed PMID: 30826147.
